# The monocyte-macrophage axis in the intestine

**DOI:** 10.1016/j.cellimm.2014.03.012

**Published:** 2014-09

**Authors:** Calum C. Bain, Allan McI Mowat

**Affiliations:** Centre for Immunobiology, Institute of Infection, Immunity and Inflammation, University of Glasgow, United Kingdom

**Keywords:** Intestine, Macrophages, Monocytes, Homeostasis, Inflammation

## Abstract

Macrophages are one of the most abundant leucocytes in the intestinal mucosa where they are essential for maintaining homeostasis. However, they are also implicated in the pathogenesis of disorders such as inflammatory bowel disease (IBD), offering potential targets for novel therapies. Here we discuss the function of intestinal monocytes and macrophages during homeostasis and describe how these populations and their functions change during infection and inflammation. Furthermore, we review the current evidence that the intestinal macrophage pool requires continual renewal from circulating blood monocytes, unlike most other tissue macrophages which appear to derive from primitive precursors that subsequently self-renew.

## Highlights

• Mϕ are involved in gut homeostasis and the pathogenesis of intestinal inflammation.
• Resident and proinflammatory intestinal Mϕ both derive from Ly6C^hi^ blood monocytes.
• Local environmental factors guide monocyte differentiation in the gut mucosa.
• Monocyte differentiation is disrupted by inflammation resulting in the accumulation of proinflammatory cells.

## 1 Introduction

The intestine encounters more antigen than any other part of the body and therefore it is no surprise that it is home to the largest compartment of the immune system. Mononuclear phagocytes (MPs), including both dendritic cells (DC) and macrophages (mϕ), play a central role in discriminating harmful from harmless antigens. They initiate and sustain protective immune responses mounted towards pathogenic organisms, but also ensure that local and systemic tolerance is generated in response to innocuous antigens. When tolerance against dietary proteins and the resident commensal microbiota breaks down, this can lead to chronic inflammatory disorders such as coeliac disease and Crohn’s disease; MPs are also implicated in these processes [1]. Understanding whether these somewhat paradoxical roles are carried out by the same MP, or if independent, functionally distinct subsets of MP exist is important for the development of new therapies for the treatment of inflammatory bowel disease (IBD) and other conditions.

In this review, we will discuss how intestinal macrophages (mϕ) contribute to these processes, highlighting how they develop from classical monocytes and showing how their function and fate alters depending on the presence or absence of inflammation.

## 2 Mononuclear phagocyte heterogeneity in the intestine

Defining the nature and origins of individual subsets of intestinal MPs has been complicated by the fact that functionally distinct populations have overlapping phenotypes. For instance, although DC are identified traditionally by their expression of CD11c and MHCII, it is now clear that multiple cell types express CD11c in the mucosa, including mϕ and eosinophils [2]. Furthermore, resident gut mϕ express high levels of MHCII [3], [4], [5], meaning that a panel of different markers is needed to discriminate these cells properly (see below). The importance of identifying these cells precisely is underlined by the fact that DC and mϕ fulfil quite distinct functions in intestinal immune responses. By definition, DCs migrate constitutively in a CCR7-dependent manner to the draining lymph node, where they interact with and cause differentiation of recirculating naïve T cells. On the other hand, mϕ are sessile, tissue resident cells whose principal role is to clear and degrade debris or pathogens, with little or no ability to prime naïve T cells [6].

Recent work from ourselves and others has shown that a combination of surface markers is needed to tease apart DC and mϕ amongst the MP populations found in normal intestinal lamina propria (LP) (see Table 1). F4/80 and CD64 (the high affinity FcRγ1) are particularly useful in this respect, as CD11c^+^MHCII^+^ MPs expressing these markers have been shown to be resident mϕ, with high phagocytic activity, little ability to prime naïve T cells and being absent from intestinal lymph [6], [7], [8], [9]. In contrast, CD11c^+^MHCII^+^ MPs that lack expression of F4/80 and CD64 are found in afferent lymph, prime naïve T cells and are non-phagocytic [7], [8], [9]. Their identity as *bona fide* DC is confirmed by their expression of the DC-specific transcription factor Zbtb46 and their dependence on flt3L for their development, properties not shared by the CD64^+^ mϕ [6], [9], [10]. The migratory F4/80/CD64^-^ MPs can be further subdivided into four subsets on the basis of CD103 and CD11b expression, with each subset displaying functional specialisation in terms of T cell polarisation [9] (reviewed in [11]). Importantly, these studies also show that CD103 expression alone cannot be used to distinguish DC from mϕ, as has sometimes been assumed. Similar cautions must be applied to the interpretation of CX3CR1 expression. Although it is now clear that all intestinal MPs expressing high levels of CX3CR1 are F4/80^+^CD64^+^ mϕ and most mature intestinal mϕ are CX3CR1^hi^, intermediate levels of CX3CR1 (CX3CR1^int^) can be found on cells of both the DC and mϕ lineage [6], [7], [9], emphasising the need for additional markers in distinguishing these cell types (see Table 1).

**Table 1:** Surface marker expression by monocytes, macrophages and dendritic cells in the intestinal mucosa.

	Newly extravasated monocytes	Mature macrophages	Dendritic cells
CD11b	+	+	+/−⁎
CD11c	−	++	+++
CD14	+	++	−
CD64	Low	+++	−
CD103	−	−	+/−⁎
CD172a	+	+	+/−⁎
F4/80	Low	+++	−
MHCII	−	+++	+++
Ly6C	+++	−	−
CX3CR1	++	+++	+/−⁎

⁎ These markers define functionally distinct dendritic cell subsets with specific transcription factor requirements.

## 3 Functions of macrophages in the steady state mucosa

The primary function of intestinal mϕ is to act as effector cells of the innate immune system. Their close association with the epithelial monolayer, coupled with their high phagocytic and bactericidal activity, means they are ideally positioned to capture and destroy any material breaching the epithelial barrier. They may also be involved in clearing effete epithelial cells [12] and in tissue remodelling [13]. However, unlike mϕ in other tissues, ingestion of bacteria or exposure to stimuli such as TLR ligands does not trigger pro-inflammatory responses by mucosal mϕ, which display remarkable anergy to such stimulation [7], [14]. Despite being avidly phagocytic, they also lack respiratory burst activity [15] or generation of nitric oxide [16]. Nevertheless, these cells are not completely inert and indeed, they show features of having been activated by their local environment, including high levels of MHCII expression, constitutive production of TNFα and a foamy, vacuolated cytoplasm [7], [17], [18], [19]. Intestinal mϕ also play an active role in maintaining the integrity of the epithelial barrier through the production of prostaglandin E2, which promotes the proliferation and survival of epithelial progenitors [20]. Thus, they appear to exist in a balanced state in which partial activation has occurred, but inflammation is prevented.

## 4 Intestinal macrophages and adaptive immunity

CX3CR1^hi^ mϕ in LP take up orally administered protein antigens efficiently [21] and because of their expression of high levels of MHCII, intestinal mϕ are frequently included amongst the “antigen presenting cells” (APC) of the mucosa [22]. Indeed it has been proposed that they play a specific role in driving the differentiation of antigen-specific FoxP3^+^ regulatory T cells (Treg) from naïve CD4^+^ T cells in the intestine [23] and anatomical differences in the numbers of Treg in different parts of the intestine correlate with the numbers of mϕ [24]. However under steady state conditions, intestinal mϕ cannot activate naïve CD4^+^ T cells *in vitro* and do not migrate to the MLN [6]; furthermore naïve CD4^+^ T cells are essentially absent from the normal mucosa [25]. Thus it is unlikely that mϕ can be involved in the initial priming of Treg. Instead IL10 production by gut resident mϕ may facilitate the secondary expansion and maintenance of FoxP3^+^ Treg that have migrated there after initial priming in the MLN [26], [27]. Intestinal mϕ may play a similar role in the maintenance of other types of mucosal T cells, with microbiota-driven production of IL1β by mucosal mϕ having been shown to assist the development of Th17 cells [28]. Whether these processes require cognate interactions between antigen specific T cells and mϕ *in vivo* remains to be determined.

## 5 Origins of steady state intestinal macrophages

### 5.1 Circulating monocytes and tissue macrophage homeostasis

The traditional view of the mononuclear phagocyte system (MPS) is that monocyte precursors develop in the bone marrow (BM), with mature monocytes then entering the circulation and migrating into the organs of the body to replenish tissue macrophages [29]. In mice, monocytes arise from the common macrophage and dendritic cell progenitor (MDP) [30], [31] through a common monocyte progenitor (cMoP) [32]. They express the CSF1R (CD115) and two subsets can be identified on the basis of Ly6C (or Gr-1) expression [33]. The larger subset is Ly6C^hi^ (Gr-1^+^) and expresses high levels of CCR2 and CD62L, but low levels of CX3CR1 [33]. Although these Ly6C^hi^ monocytes were originally termed “inflammatory”, given their readiness to enter inflamed tissues, they are now referred to as ‘classical’ monocytes [34]. Ly6C^hi^ monocyte egress from the BM is dependent on the CCR2 chemokine receptor and mice deficient in CCL2 or CCR2 have essentially no circulating monocytes, as well as showing defective recruitment of mϕ during inflammation [35], [36]. The smaller subset of Ly6C^lo^CX3CR1^+^ monocytes expresses lower levels of CCR2 and CD62L and was originally proposed to be a distinct lineage of monocytes that replenished steady state macrophage populations [33]. However current evidence indicates that Ly6C^lo^ monocytes are the progeny of CSF-1 dependent maturation of Ly6C^hi^ monocytes and they have little or no ability to emigrate from the bloodstream into tissues [37], [38]. As such, their primary function is now believed to be in the maintenance of the vasculature, including the disposal of apoptotic endothelial cells through the recruitment of neutrophils [39], [40]; they are now referred to as ‘patrolling’ or ‘non-classical’ monocytes [34].

Human CD115^+^ monocytes have also been segregated into subsets based on their expression of the LPS co-receptor CD14 and CD16 (FcγRIII) [41]. Whereas CD14^hi^ CD16^−^ monocytes express CCR2 and are the equivalent of classical Ly6C^hi^ murine monocytes, CD14^lo^CD16^+^ monocytes lack CCR2 expression and are homologous to non-classical monocytes [34]. As in mice, the human monocyte subsets appear to be related to each another developmentally, with CD14^hi^CD16^-^ monocytes maturing into CD14^lo^CD16^+^ ‘non-classical’ monocytes through a CD14^+^CD16^+^ intermediary. Gene expression analysis has revealed that there is a high degree of conservation between the homologous subsets in mouse and man [42].

### 5.2 Generation of tissue macrophages from foetal precursors

More recently, it has been suggested that blood monocytes play little or no role in the homeostasis of resident tissue mϕ. Rather, it is proposed that these are derived from progenitors arising in the yolk sac (YS) and/or foetal liver (FL) that seed tissues during embryonic development and are then maintained in adult life by self-renewal *in situ*. Using a variety of lineage tracking techniques and cell kinetic approaches, this process has been shown to be the origin of microglia in the central nervous system (CNS) [38], [43], [44], liver Kupffer cells [38], [44], lung alveolar mϕ [38], [45], mϕ of the peritoneal cavity [46] and Langerhans cells in the epidermis of the skin [38], [44], [47], [48]. By extension, it is often assumed that similar mechanisms apply to mϕ in all other healthy tissues [49]. However our recent work indicates that the intestinal mϕ pool is an exception to this hypothesis, by requiring continuous replenishment by blood Ly6C^hi^ monocytes [7]. Varol and colleagues were the first to show that classical Ly6C^hi^ monocytes could give rise to CD103^−^CD11b^+^CX3CR1^+^ MP in the mucosa of myeloid cell depleted hosts and this was confirmed by subsequent work [22], [50], [51]. Although the cells derived from Ly6C^hi^ monocytes were initially considered to be CD103^−^ DC, it is now clear that most actually belong to the mϕ lineage and more recently, it has been shown directly that Ly6C^hi^ monocytes give rise to F4/80^+^ CD64^+^ CX3CR1^hi^ resident mϕ in steady state intestine [7], [8].

The recruitment of Ly6C^hi^ monocytes to the adult mucosa is dependent on CCR2, as mice lacking either CCR2 or its ligand CCL2 have markedly reduced intestinal mϕ pools [7], [52]. That this active recruitment needs to continue throughout life is shown by the fact that acute interruption of the CCL2-CCR2 axis in CCR2-DTR mice causes a rapid loss of intestinal mϕ [53]. Furthermore, intestinal CD64^+^ mϕ derive almost exclusively from WT BM in WT:CCR2^−/−^ mixed BM chimeric mice [8]. Steady state intestinal mϕ also do not divide *in situ* [7], [54], indicating they unlikely to self-renew and underlining the requirement for an external top-up supply.

The cellular source of CCL2 which drives monocyte recruitment is unknown, as are mechanisms responsible for its production in the mucosa. Although exposure to the commensal microbiota would seem an obvious candidate, studies of the size of the intestinal mϕ population in germ free mice have produced conflicting results [17], [55], [56]. There have also been no direct studies of monocyte recruitment into the mucosa of GF or antibiotic treated mice.

Finally, it is important to note that CCR2 may not be the only recruitment mechanism, as intestinal mϕ are not entirely absent in steady state CCR2^−/−^ and CCL2^−/−^ mice [7], [52]. Other mediators which have been implicated by *in vitro* studies include TGFβ and IL8 [18], [54], but the role of these and other mediators has not been addressed *in vivo*. As noted above, it seems unlikely that the Ly6C^lo^ population of monocytes may be involved in the homeostasis of intestinal mϕ. Although they can give rise to CD11c^+^ CX3CR1^+^ myeloid cells in Peyer’s patches (PP) [51], Ly6C^lo^ monocytes have very limited ability to enter non-lymphoid tissues and adoptive transfer approaches have failed to find them in recipient intestinal mucosa [7], [51]. Furthermore, intestinal mϕ numbers appear to be normal in CX3CR1-deficiecnt mice [26], which would not be anticipated if Ly6C^lo^ monocytes were involved, due to their dependence on the CX3CL1-CX3CR1 axis for survival [57], [58]. Nevertheless, given the high expression of CX3CL1 by epithelial and goblet cells and the apparent requirement for CX3CR1 in formation of transepithelial dendrites by CX3CR1^+^ MPs [58], it remains possible that this chemokine pathway could be involved in localisation of intestinal mϕ towards the epithelial barrier after their arrival in the mucosa.

Together these findings indicate that the principal source of the resident intestinal mϕ pool is CCR2-dependent recruitment of Ly6C^hi^ monocytes. However whether mϕ derived from primitive progenitors also exist in the intestine alongside Ly6C^hi^ monocyte-derived mϕ has never been formally tested.

## 6 Monocyte differentiation in the steady state mucosa

As discussed above, Ly6C^hi^ monocytes were originally referred to as ‘inflammatory’ monocytes on the grounds that they migrated efficiently into inflamed tissues. Together with the fact that Ly6C^hi^ monocytes respond vigorously to TLR ligation and other stimuli *in vitro* [59], it was therefore initially surprising to find that they appeared to be the source of a profoundly anti-inflammatory pool of mϕ. The explanation for this comes from experiments showing that very soon after entry to the mucosa, Ly6C^hi^ monocytes begin to undergo a process of local differentiation that results in the generation of mature mϕ. This occurs via a series of short-lived CX3CR1^int^ intermediaries, which first acquire MHCII before losing Ly6C expression and upregulating F4/80, CD64 and CX3CR1 [7]. Within the space of 4–5 days, they have acquired the F4/80^hi^CD64^+^MHCII^+^CD11c^+^CX3CR1^hi^ phenotype typical of resident intestinal mϕ [7]. The phenotypic differentiation continuum of monocytes is paralleled by progressive acquisition of the typical functions of resident intestinal mϕ, with increasing production of IL10, enhanced phagocytic activity, acquisition of scavenger receptors and the development of unresponsiveness to TLR ligation [7]. These processes appear to be unique to the intestine, as although Ly6C^hi^ monocytes enter other tissues such as the lung, they do not undergo the same phenotypic changes [60]. Interestingly however, a similar developmental series of Ly6C^hi^ monocytes has recently been identified in skin [61], suggesting that the origins of tissue mϕ may need to be re-examined, especially those at body surfaces where constant immune surveillance is needed.

The factors that influence monocyte differentiation in the intestinal mucosa remain to be identified with certainty, and it is quite likely that more than one factor may be needed to explain all aspects of the fully adapted profile. The development of mϕ in the steady state gut mucosa is dependent on CSF1R, with essentially all mϕ being derived from WT BM in WT:CSF1R^−/−^ mixed BM chimeric mice [62] and their numbers are severely depleted by administration of anti-CSF1R antibody *in vivo* [63]. Whether this is due to the actions of CSF1 or IL34, the only other identified CSF1R ligand, remains unclear. Although epidermal Langerhans cells and microglia of the CNS have been shown to depend on the IL34-CSF1R axis [64], [65], the impact of IL34-deficiency on the intestinal mϕ compartment has never been examined directly. This CSF1R-dependency contrasts with the resident mϕ in some other tissues that are derived from primitive precursors such as alveolar mϕ, whose development is dependent on CSF2 [46], [66]. However CSF1R signalling alone is unlikely to account for all aspects of intestinal mϕ development, as it is required for the development of other monocyte-derived mϕ [62], [63], whereas adoptively transferred monocytes generate entirely distinctive mϕ in the mucosa. Thus the local environment of the intestinal mucosa must play a definitive role in local mϕ development.

Most work on this topic has concentrated on the inability of intestinal mϕ to respond appropriately to inflammatory stimuli. This is due at least in part to the constitutive production of high levels of IL10 and colonic mϕ from IL10-deficient mice or mice with LysM driven deletion of the IL10R signalling molecule STAT3 respond robustly to TLR stimulation with proinflammatory cytokine production [17], [55], [67], [68]. Moreover, spontaneous colitis develops in mice in which the IL10-IL10R regulatory axis has been disrupted [69], [70] and IL10R deficiency leads to early onset, fulminant IBD in man [71]. Colitis development in the absence of IL10 signalling appears to be directly attributable to inappropriate mϕ behaviour directed toward commensal bacteria, as rendering mϕ unresponsive to TLR ligands through the deletion of the key TLR adaptor molecule MyD88, prevents colitis development in the IL10-deficient mouse [68]. A role for IL10 in determining other aspects of monocyte differentiation in the mucosa remains to be explored, although it is known that the expression of the scavenger receptor (CD163) and the mannose receptor may be under control of IL10 [67].

TGFβ is a further mediator that can reproduce the TLR hyporesponsiveness of mature intestinal mϕ in monocytes or BM derived mϕ and *in vivo*, this could be derived from components of the intestinal extracellular matrix or epithelial cells [18], [72]. Although TGFβ has also been reported to induce CX3CR1 expression by microglia in the brain [73], as yet there is no evidence that it can account for the other features of resident intestinal mϕ such as IL10 production, MHCII expression or scavenger activity.

In addition to an effect on the recruitment of monocytes to the mucosa, the microbiota may also influence their subsequent differentiation, as mϕ in the germ free intestine produce less IL10 [17]. There has been considerable interest in the idea that ligation of TLR receptors by the microbiota could promote a feedback inhibition pathway, similar to the well known phenomenon of “endotoxin tolerance”, in which exposure to a single TLR ligand such as LPS prevents subsequent responses to the same agent. Whether this accounts for the anti-inflammatory properties of resident intestinal mϕ is unclear, as is the nature of any TLR involved. For instance the defective IL10 production seen in GF mice is not replicated in MyD88^−/−^ mice [17], indicating some specificity to the phenomenon. Notably, the hyporesponsiveness does not involve loss of receptors such as TLR [7], [18], although there may be downregulation of associated adapter and signalling molecules such as CD14, MyD88, TRAF-6, MD2, TRIF and IRAK1 [14], [18], [19]. In parallel, mature intestinal mϕ upregulate mechanisms that interfere with TLR signalling and/or NF-κB activation, including IRAK-M and IkBNS [18], [19], [67]. Although some of these inhibitory processes may be driven by IL10, other mechanisms are clearly involved. It is also important to note that the hyporesponsiveness of intestinal mϕ does not just apply to TLR ligation, but affects reactivity to a wide variety of stimuli including NOD ligands, intact bacteria, phagocytosed particles and γIFN amongst others [14]. The role of other pattern recognition receptors (PRR) in the effects of the microbiota has not been investigated, and the full range of intestinal mϕ functions in GF mice is not known.

Despite high levels of CX3CR1 expression being one of the most characteristic features of resident intestinal mϕ and the fact that its ligand CX3CL1 is produced by epithelial cells [58], the exact role CX3CR1 plays in controlling mϕ function is unclear. The CX3CL1-CX3CR1 axis has been shown to control the ability of CX3CR1^+^ MPs to sample luminal contents through the formation of transepithelial dendrites (TEDs) [58], [74], [75]. However the physiological significance of TED formation remains unclear, as different groups have found them in different parts of the intestine, and have reported them to be both TLR dependent and independent [76]. Others have failed to find them at all in the steady state intestine [77].

As noted above, the CX3CL1-CX3CR1 axis appears to be required for IL10 production by intestinal mϕ [26], although the mechanisms for this are unclear and other studies of CX3CR1 function in intestinal mϕ have produced conflicting results. For instance, CX3CR1-deficient mice have been shown both to be protected [56], [58] and more susceptible to experimental colitis [78]. Thus further investigation of the CX3CR1-CX3CL1 axis is required to elucidate its involvement in the control of mϕ function in the intestine.

The induction of MHCII on monocytes after their arrival in the mucosa is a particularly intriguing and as yet, unexplained phenomenon. It occurs normally in rag2^−/−^, IL10^−/−^ and γIFNR^−/−^ mice, eliminating many of the usual suspects for this phenomenon such as lymphocytes and γIFN signalling (our unpublished observations). Extravasation of Ly6C^hi^ monocytes through local vascular endothelium has also been reported to induce upregulation of MHCII by skin mϕ [60], but this has not been explored with intestinal mϕ.

Thus it is clear that multiple factors may influence monocyte differentiation in the normal mucosa, acting in concert to ensure that monocytes adapt to their unique immediate microenvironment. However the nature and mechanisms of action of these mediators remain to be defined fully.

## 7 Monocytes and macrophages in intestinal inflammation

The composition of the macrophage compartment in the intestinal mucosa changes markedly when there is disruption of tissue homeostasis by infection, inflammation or trauma. A variety of experimental models has shown that this results in the accumulation of monocytes and their proinflammatory mediators.

### 7.1 Sterile inflammation

During sterile intestinal inflammation, such as that induced by feeding of DSS or transfer of naïve CD4^+^ T cells into lymphopenic hosts, the normal balance between the proportions of CX3CR1^hi^ and CX3CR1^int^ cells is reversed, with intense accumulation of Ly6C^hi^ monocytes and their immediate progeny [7]. In contrast, the numbers of resident CX3CR1^hi^ mϕ change very little, because unlike under steady state conditions, elicited monocytes do not mature fully into anti-inflammatory CX3CR1^hi^ mϕ. Instead they remain as CX3CR1^int^ cells that retain their enhanced production of proinflammatory cytokines (e.g. TNFα, IL6, IL12 and IL23), iNOS and remain responsive to TLR stimulation [7], [19], [79]. Conversely, the remaining resident CX3CR1^hi^ mϕ retain their anti-inflammatory signature, despite the presence of active inflammation [7], [19].

There are multiple lines of evidence to suggest that Ly6C^hi^ monocytes and their derivatives are directly pathogenic in chemically induced colitis. Firstly, monocytopenic CCR2-deficient mice are less susceptible to DSS-induced colitis [80]. Second, administration of an anti-CCR2 depleting antibody, which is presumed to target Ly6C^hi^ monocytes selectively, ameliorates colitis and correlates with lower levels of IL6 and IL1β in colonic tissue [19]. Finally, DSS-induced colitis is severely reduced in mice in whom Ly6C^hi^ monocytes are deficient in TNFα production [51]. As well as a direct proinflammatory role, Ly6C^hi^ monocytes and their progeny may orchestrate the recruitment of other immune effector cells. For instance, Ly6C^hi^ monocyte-derived CCL11 is responsible for the recruitment of CCR3^+^ eosinophils during DSS-induced colitis [81]. Thus during sterile inflammation, Ly6C^hi^ and their derivatives appear to be overtly proinflammatory and as a result pathogenic.

Although most studies have focussed on the idea that the infiltration of the mucosa by inflammatory cells is driven by CCR2 dependent recruitment of blood monocytes, recent work suggests that intestinal neutrophils may also accumulate in T cell dependent colitis via local differentiation of granulocyte-monocyte progenitors present in the mucosa [82]. Whether this additional mechanism could also apply to monocytes and mϕ remains to be examined.

### 7.2 Intestinal infection

CX3CR1^int^ cells and Ly6C^hi^ monocytes also accumulate in vast numbers in the intestinal mucosa in response to pathogenic infections such as *Toxoplasma gondii* [83], [84], *Citrobacter rodentium* [85] and *Salmonella typhimurium* [12].

Oral inoculation of certain mouse strains with the protozoan parasite *T. gondii* results in acute ileitis and the accumulation of innate effector cells. Toxoplasmosis is lethal in CCR2- and CCL2-deficient mice and can be rescued by the adoptive transfer of CCR2-competent Ly6C^hi^ monocytes [83], suggesting a protective role for Ly6C^hi^ monocytes and their progeny. As in DSS colitis, the Ly6C^hi^ monocytes elicited by *T. gondii* infection produce the proinflammatory cytokines TNFα and IL12, as well as reactive nitrogen species [83]. Activation of monocytes in this infections requires TLR-dependent recognition of PAMPs, as it is abrogated in MyD88^−/−^ animals [86]. As well as being recruited by the classical CCL2-CCR2 axis, Ly6C^hi^ monocytes can be attracted to the *T gondii* infected mucosa in a CCR1-dependent manner by CCL3 derived from NKp46^+^ innate lymphoid cells (ILC) [84]. Manipulation of the CCL3-CCR1 axis by gene deletion or antibody blockade results in fewer infiltrating Ly6C^hi^ monocytes and reduced inflammation, but higher parasite burden, again supporting the idea that these cells are directly involved in parasite clearance.

Infection with *C. rodentium* is used widely as a model of enteropathogenic and enterohaemorrhagic *Escherichia coli* infection in man [85]. CCL2- and CCR2-deficient mice show delayed clearance of *C.* *rodentium* due to failure to recruit CD11b^+^ cells, implicating Ly6C^hi^ monocytes in the protective response [85]. It is believed that CCL2 production by stromal cells following NOD2-dependent recognition of *C. rodentium* is the principal mechanism responsible for the recruitment of CD11b^+^ cells to the colonic mucosa [85]. Interestingly however, DT-mediated ablation of the entire monocyte/macrophage compartment using LysM-cre x CSF1R-STOP-DTR mice does not alter the susceptibility to or ability to clear *C. rodentium* infection [87]. In contrast, mice lacking migratory DC due to deficiency in flt3L or ablation of Zbtb46^+^ cells, succumb to lethal infection [10], [87], consistent with the known role for DC in driving protective Th17 responses against *C. rodentium* [85]. Thus monocytes/mϕ contribute to, but are dispensable for immunity towards *C. rodentium*.

Infection with *Salmonella* species has also demonstrated a role for monocyte-derived cells in host protection. Infection of PP by *S.* *typhimurium* results in the accumulation of highly bactericidal Ly6C^hi^ CD68^+^ MP that express iNOS and MHCII, and produce TNFα [88]. Studies using mutant forms of *S. typhimurim* that lack a functional type III secretion system (e.g. InvG), have shown that Salmonella species can also enter the LP independently of PP [89]. This ‘alternative pathway’ relies on bacterial uptake by CD11c^+^CX3CR1^+^ MP in a MyD88-independent fashion [89]. More recent studies have shown that uptake likely involves CX3CR1^hi^ resident mϕ and elicited CX3CR1^int^ cells [12], many of which are likely to be derivatives of Ly6C^hi^ monocytes.

Although not yet studied in any detail, our own preliminary experiments suggest that chronic infection with *Helicobacter hepaticus* also leads to accumulation of Ly6C^hi^ monocytes and CX3CR1^int^ early stage mϕ which peaks at the same time as the protective γIFN/IL17 T cell response [90] (and our unpublished observations). Together these results underline the possible role of Ly6C^hi^ monocytes in protective immunity during intestinal infection with bacterial and intracellular pathogens.

Whether blood monocytes contribute at all to Th2-type immunity and to the generation of the associated “alternatively activated” mϕ is more questionable. Depletion of circulating monocytes does not affect the numbers or activity of mϕ during pleural infection with the filarial nematode *Litomosoides sigmondontis* [91]. Instead, a population of alternatively activated mϕ expands through *in situ* self-renewal. However, genetic deletion of CCL2 renders normally resistant C57Bl/6 mice susceptible to intestinal *Trichuris muris* infection [92], the natural mouse model of human *Trichuris trichura* infection, one of the most prevalent intestinal helminth infections. Similarly depletion of monocytes and mϕ by administration of clodronate liposomes leads to reduced clearance of *Heligomosomoides polygyrus* from the intestine [93]. This discrepancy between intestinal and pulmonary helminth infection could be consistent with the different origin of tissue mϕ in these tissues under normal conditions and further studies are required to fully elucidate the role of Ly6C^hi^ monocytes during Th2 immunity.

## 8 Monocytes/macrophages and adaptive immunity during inflammation

Several reports have suggested that monocytes and their progeny may accumulate in the draining MLN and contribute to the generation of effector T cells there during intestinal inflammation [8], [19], [94]. However the nature of the cells and how they get to the MLN is controversial.

During DSS-induced colitis and T-cell transfer colitis, it has been proposed that Ly6C^hi^ monocytes can give rise to a population of DC [17], [19], which migrate from the mucosa to the MLN via afferent lymphatics [19]. However other studies have challenged this concept of monocyte-derived DC, by showing that Ly6C^hi^ monocytes in the mucosa give rise only to the mϕ lineage even during overt inflammation [7], and indicate that any monocyte-derived cells that appear in the MLN arrive via the bloodstream. In support of this idea, the Ly6C^hi^ monocyte-derived MP that accumulate in the MLN during T cell transfer colitis lack CCR7 [94] and this process occurs in the absence of CCR7 [8]. Recently, it has been suggested that some of the normally tissue resident CX3CR1^hi^ mϕ in intestinal mucosa may acquire migratory capacity during infection with *S. typhimurium* and may contribute to initiation of the specific immune response in MLN [95]. However, with the discovery of *bona fide* CD103^-^CD11b^+^ DC that also express CX3CR1 at intermediate levels [7], [9], the identity and origin of the CX3CR1^+^ MP that capture and transport Salmonella to the MLN need to be clarified.

It seems highly likely that Ly6C^hi^ monocytes and their derivatives may be able to interact with T cells in the inflamed mucosa itself. Consistent with this idea, cells of the monocyte/mϕ lineage appear to be required to maintain IFNγ-producing and IFNγ^+^ IL17^+^ T cells in the mucosa during *C. rodentium* infection through their local production of IL12 [87]. In addition, gut resident CX3CR1^hi^ macrophages that retain their anti-inflammatory properties during active colitis may act to suppress effector T cell proliferation [96].

## 9 A regulatory role for Ly6C^hi^ monocytes?

Although Ly6C^hi^ monocytes and their progeny have generally been seen as proinflammatory effector cells, recent work has suggested that they may have a dual role during *T. gondii*-induced intestinal inflammation. Grainger and colleagues [97] have demonstrated that as well as exhibiting potent parasite killing activity and production of proinflammatory mediators, Ly6C^hi^ monocytes possess regulatory properties such as the production of IL10 and expression of arginase. Moreover, these elicited Ly6C^hi^ monocytes can inhibit the pathogenic effects of neutrophils through COX-2-dependent production of PGE2 [97]. Indeed this may explain earlier observations of enhanced neutrophil accumulation in the mucosa of *T. gondii* infected CCR2-deficient animals [83]. Thus Ly6C^hi^ monocytes appear to be Janus-like, controlling both parasite clearance and restoration of homeostasis by regulating the recruitment and activity of other innate effector cells. Whether this regulatory role is specific to intestinal Ly6C^hi^ monocytes in *T. gondii* infection, or if these characteristics have been overlooked in other models of infection and inflammation remains to be tested. For example, although CD11b^+^ cells display both proinflammatory (TNFα, iNOS, IL12p40) and regulatory characteristics (IL10, arginase, Ym-1 expression) during *C. rodentium* infection [85], in this instance resident mϕ and elicited cells were analysed as a single entity, making it unclear which characteristics belong to which population.

## 10 Monocytes/macrophages during resolution of inflammation

The composition of the mϕ pool also changes markedly during the resolution of intestinal inflammation. In mice, the CX3CR1^int^ compartment contracts to homeostatic levels, which is paralleled by a contraction in neutrophil numbers [19]. Whether any elicited Ly6C^hi^ monocytes persist and convert into anti-inflammatory CX3CR1^hi^ mϕ, or if they are cleared through local apoptosis as in other tissues [98] remains unclear. Importantly, the contribution of intestinal mϕ to the restoration of homeostasis is dependent on the TGFβ−TGFβR axis, as there is delayed resolution of DSS-induced colitis when mϕ are unresponsive to TGFβ [13]. In view of the potential importance of understanding the mechanisms of tissue repair and healing for treating chronic inflammatory disease, more studies are warranted on how mϕ can adapt to different conditions.

## 11 Monocytes in human gut

Consistent with mouse studies, mϕ are one of the most abundant leucocytes in the human intestinal mucosa, where they accumulate close to the epithelial layer [7]. Although often identified by their expression of CD33 and CD13 [4], [99], [100], recent studies have shown that there is sufficient conservation of surface marker expression between mouse and man to allow more direct comparisons to be made. Thus mϕ dominating the healthy human ileal mucosa express CD64, MHCII and the scavenger receptor CD163 [7], as well as CD68 and CD14 [4], [7], [18]. Whereas resident mϕ express low levels of CD14, a smaller population of CD14^hi^ mϕ is also present in the healthy mucosa [7]. These CD14^hi^ cells are heterogeneous for expression of MHCII, CD163 and CD209, as well as CD11c, suggesting that they may represent recent CD14^hi^ monocyte emigrants, akin to the continuum of CX3CR1^int^ cells seen in the mouse intestine [7]. Interestingly, mϕ resident in the human small intestine uniformly express high levels of MHCII [14], whereas those in the colonic mucosa have been suggested to express low levels of MHCII [4]. This dichotomy is not seen in mouse and thus the anatomical location of mϕ may impact upon their phenotype selectively in humans.

During intestinal inflammation such as that associated with Crohn’s disease and ulcerative colitis, CD14^hi^ monocyte-derived cells have been shown to accumulate [7], [99], [101], [102]. These recruited CD14^hi^ cells display enhanced production of TNFα, IL1β and IL6 [102] and respiratory burst activity [15], as well as responsiveness to exogenous stimulation by e.g. TLR ligands [99], [101]. However recruited monocytes may behave differently in CD compared with UC, for instance only those in the CD inflamed mucosa produce IL23, consistent with Th1/Th17 skewing in this condition [98], [99]. Elegant studies using transfer of radiolabelled autologous blood monocytes have demonstrated that these CD14^hi^ monocytes derive from circulating monocytes rather than proliferation of gut resident cells [103], while elevated levels of the monocyte chemoattractants CCL2 and CCL4 are found in the mucosa of IBD patients [104]. Similar to Ly6C^hi^ monocytes infiltrating the inflamed mouse mucosa, CD14^hi^ monocyte/macrophages in the UC inflamed mucosa have been shown to produce chemokines to attract other effector leucocytes, such as eosinophils through their production of CCL11 [100]. Whether these infiltrating CD14^hi^ cells exhibit also exhibit regulatory characteristics remains to be explored. Thus the behaviour of monocytes and mϕ in human and mouse intestine seems to follow analogous processes in both steady state and in inflammation, suggesting animal models remain a valuable means of exploring these processes.

## 12 Concluding remarks

Much progress has been made recently into understanding the role of mϕ in the intestine under physiological conditions, as well as when the steady state is disrupted by inflammation and/or infection. In both settings, mϕ of the gut wall derive from blood monocytes that enter the mucosa and mature locally, with their fate dictated by the cues received from their immediate environment. Thus future work must set out to identify the factors that promote or disrupt monocyte differentiation as these may hold promise as new therapeutic targets for the treatment of IBD.
